# Healthcare professionals’ perspectives on the barriers and enablers of cancer clinical trial participation among Arabic-speaking cancer patients in Australia: a qualitative study

**DOI:** 10.1186/s13063-026-09815-z

**Published:** 2026-06-11

**Authors:** Rayan Saleh Moussa, Layla M Edwards, Michael Camit, Christine Jeyachandran, Tim Luckett, Ben Smith, Bernadette Brady, Slavica Kochovska, Charbel Bejjani, Maria Gonzalez, Nadine El-Kabbout, Arwa Abousamra, Amin Gadalla, Iman Zakhary, Azhar Matti, Randa Kattan, Verena Wu, Wei Chua, Karen Wong, Shalini Subramaniam, Gemma McErlean, Balwinder Sidhu, Matthew Jennings, Sandra Avery, Deme Karikios, Frances Boyle, Wafa Trad, Weng Ng, Stephen Della-Fiorentina, Martin Hong, Udit Nindra, Sally Fielding, Meera Agar, Abhijit Pal

**Affiliations:** 1 Improving Palliative, Aged and Chronic Care through Clinical Research and Translation (IMPACCT), Faculty of Health, University of Technology Sydney, Sydney, NSW Australia; 2 South Western Sydney Local Health District, Health Literacy-Multicultural Services, Sydney, NSW Australia; 3 The Daffodil Centre, The University of Sydney, and Cancer Council NSW, Sydney, NSW Australia; 4 South Western Sydney Local Health District, Sydney, NSW Australia; 5 Australian Centre for Cancer Equity, Sydney, NSW Australia; 6 College of Medicine & Public Health, Flinders University, Adelaide, SA Australia; 7 Implementation to Impact, School of Public Health, University of New South Wales, Sydney, NSW Australia; 8 Arab Council Australia, Sydney, NSW Australia; 9 University of New South Wales, Sydney, NSW Australia; 10 Western Sydney University, Sydney, NSW Australia; 11 Centre for Research in Nursing and Health, St George Hospital, South Eastern Sydney Local Health District, Sydney, NSW Australia; 12 School of Nursing, University of Wollongong, Wollongong, NSW Australia; 13 Nepean Cancer and Wellness Centre, Nepean Hospital, Nepean Blue Mountains Local Health District, Kingswood, NSW Australia; 14 Sydney Medical School, University of Sydney, Sydney, NSW Australia; 15 Illawarra Shoalhaven Local Health District, Wollongong, NSW Australia

**Keywords:** Health equity, Diversity and inclusion, Clinical trials, Participation, Culturally and ethnically diverse communities/minoritised communities, Inclusive research, Cancer/oncology, Arabic-speaking/communities

## Abstract

**Background:**

Culturally and ethnically diverse communities are underrepresented in cancer clinical trials, further contributing to existing health inequities. Research often overlooks how upstream social determinants of health (SDOH) might contribute to these inequities, instead focusing on individual-level barriers. Arabic is the third most spoken language in Australia and is the focus of this study. This study aims to explore how upstream SDOH influence clinical trial participation among Arabic-speaking cancer patients (ASCPs), as perceived by healthcare professionals (HCPs).

**Methods:**

A qualitative study comprising virtual focus groups with HCPs with experience recruiting ASCPs into clinical trials. Participants were recruited via professional networks and social media using snowball sampling. The Sociocultural Framework for Health Service Disparities was used to identify perceived barriers and enablers to cancer clinical trial participation relating to structural, healthcare provider, and environmental factors. Focus groups were analysed using the Best Fit approach by two independent coders.

**Results:**

Fourteen participants were recruited (ten medical specialists, two nurses, one clinical trial coordinator and one researcher). Structural and healthcare provider barriers centred on English-language proficiency. Structural barriers included limited consultation time, restrictive trial eligibility criteria, lack of translated study materials, and a lack of workforce diversity. Language discourse reduced HCPs’ confidence in patient understanding of trial information and consequently the informed consent procedure. By advocating for translated materials, booking longer consults, and engaging interpreters, HCPs became enablers to trial participation. Family support networks were identified as environmental enablers by facilitating trial-related conversations; however, they also acted as barriers by compromising patient autonomy in decision-making. Perceived scepticism and distress emerged as environmental barriers that shaped attitudes toward cancer diagnosis and trial participation.

**Conclusion:**

Findings highlight how upstream SDOH can create barriers to cancer clinical trial access, thereby reducing opportunities for participation among ASCPs in Australia. Although the barriers were described in terms of language, they may reflect a broader lack of cultural competency. Further research into strategies integrating enablers such as time, workforce diversity, interpreter engagement and translated resources is essential to inform inclusive healthcare systems and research design, with potential implications beyond the Arabic-speaking community.

**Trial registration:**

Ethical approval for this study was granted by the University of Technology Sydney Human Research Ethics Committee (ETH21-6506, 14/01/2022).

**Supplementary Information:**

The online version contains supplementary material available at 10.1186/s13063-026-09815-z.

## Background

Access to and effective use of healthcare is fundamental to achieving good and equitable health outcomes [1]. This is often influenced by social determinants of health (SDOH), such as age, sex, ethnicity and cultural background, education, socioeconomic status, and broader structural and social factors, which not only affect how individuals approach, utilise, and benefit from their local healthcare system, but also how healthcare systems approach and deliver care to individuals [2–4]. In multicultural countries such as the USA, UK and Australia, people from culturally and ethnically diverse communities are among those most impacted by these determinants [5–7]. Culturally and ethnically diverse communities, also referred to as culturally and linguistically diverse (CALD) in the Australian context, are groups of people who identify as coming from, or having ancestry in, countries where English is not the primary language, and who bring unique traditions, values and experiences to society [8].

Australian research has consistently shown that people who identify as culturally and ethnically diverse experience poorer healthcare access and outcomes compared to the general population [9–11]. This disparity is evident across many areas of healthcare, including cancer care. A recent scoping review identified several factors contributing to the health inequities experienced by people living with cancer from culturally and ethnically diverse communities within Australia [12]. Key barriers included language incongruence with healthcare providers and lack of interpreter services, a lack of culturally and linguistically appropriate resources such as patient information sheets about cancer and its treatments, and insufficient training and resources for healthcare providers to provide effective intercultural care [12].

Disparities affecting culturally and ethnically diverse communities extend beyond standard clinical care into medical research, where these communities are underrepresented in clinical trials, including those involving potentially life-saving cancer treatments, leading to divergent cancer outcomes [13, 14]. The lack of participant diversity in clinical trials has broader implications beyond health equity, with recent pharmacogenetic trials revealing significant differences in the metabolism, clinical effectiveness and side-effect profiles of medications amongst diverse ethnic communities [15–18]. Such evidence challenges the interpretation, validity, and generalisability of clinical research findings [19].

To date, most research, as well as national and international efforts, have primarily focused on individual-level factors such as English language proficiency, socioeconomic status, health literacy factors, cultural influences and family involvement [20, 21]. In the context of Australian cancer clinical trials, individual-level factors such as perceived lack of understanding of the health system and low English proficiency were identified as key barriers to participation in clinical research among culturally and ethnically diverse communities [14, 22, 23]. However, recent studies have emphasised that addressing individual-level barriers alone is insufficient [24, 25]. Guadamuz et al. (2024) demonstrated that environmental SDOH such as neighbourhood ethnic composition, a proxy for segregation, accounts for the majority of the inequity in clinical trial participation in the United States context [24]. In Australia, Murray et al. (2019) identified structural-level determinants that might foster inclusivity in research, including diversity in higher education, research training programmes, leadership positions, funding bodies, review processes and the regulation and implementation of policies with targets for increasing diverse representation through culturally safe practices [26].

Australia has one of the most diverse migrant populations in the world, with nearly 22.8% (5.8 million) people speaking a non-English language at home [8]. To advance clinical trial participation among culturally and ethnically diverse communities in Australia, research must move beyond individual-level factors to identify how upstream SDOH, including structural factors (e.g. healthcare organisations, their policies and study sponsors), people who work within these structures (e.g. healthcare professionals and site staff), and environmental factors (e.g. social and community context), contribute to the underrepresentation of these communities. Arabic is the third most commonly spoken language in Australia, following English and Mandarin [27]. The diversity of the Arabic-speaking population in Australia, with ancestry spanning 22 countries, alongside their prominence in Australia and globally, provided an opportunity to explore upstream SDOH influencing cancer clinical trial participation. Arabic-speaking status was used as a pragmatic indicator of linguistic access need, given the central role of language in healthcare communication and clinical trial participation. This study aimed to explore how upstream factors influence clinical trial participation among Arabic-speaking adults with cancer from the perspective of healthcare professionals (HCPs) experienced in their recruitment.

## Methods

### Study design

A qualitative design guided by an interpretative epistemology orientation was adopted to explore the meanings and experiences constructed by HCPs within their professional and cultural contexts. In line with the applied aims of the study, pragmatic elements were incorporated to support analysis relevant to equity and service improvement. This study is reported according to the COREQ (Consolidated Criteria for Reporting Qualitative Research) checklist to ensure comprehensive and transparent reporting (see Supplementary File) [28].

Data were collected through online focus groups with HCPs with experience of recruiting adult (≥ 18 years) Arabic-speaking cancer patients (hereafter referred to as *ASCPs*) to clinical trials. Semi-structured interviews were offered to HCPs unable to participate in focus groups. The focus groups and/or interviews were held online via Zoom, enabling wider reach and inclusion of potential HCPs who might otherwise be unable to attend in person. A broad definition of HCPs was applied to include a range of disciplines: medical specialists (e.g. medical or radiation oncologists, haematologists, and palliative care physicians), general practitioners, nurses, occupational therapists, physiotherapists, psychologists, social workers, speech pathologists, and professionals with relevant roles (e.g. interpreters and researchers). HCPs were eligible to participate if they worked in any healthcare setting (i.e. hospital, community, research or other) in Australia.

A semi-structured topic guide was developed collaboratively by the investigative team, informed by the study aims and existing literature on barriers and enablers to clinical trial participation. Although the guide was not formally piloted, it was reviewed by all members of the investigative team, who provided iterative feedback to refine the wording, flow, and relevance of questions before data collection commenced.

### Conceptual framework

The Sociocultural Framework for Health Service Disparities is a conceptual approach that seeks to understand and address disparities in health services experienced by different racial, ethnic, and socioeconomic groups (Pescosolido and Alegría, 2009). This framework emphasises the interplay of healthcare system structures (e.g. policies, institutions, financing arrangement), actors operating within those systems (e.g. HCPs) and environmental factors (e.g. the individuals’ social context, community conditions and lived experience) in shaping healthcare access, use, and outcomes [29–32]. This study applied the Sociocultural Framework for Health Service Disparities to guide the exploration and analysis of HCP-perceived barriers and enablers to cancer clinical trial participation among ASCPs.

### Recruitment

To maximise the diversity of professional backgrounds of participating HCPs, a multimodal strategy was used to recruit potential participants, including (i) purposive sampling, whereby an invitation email containing the registration link for the study was circulated via membership lists of Australian professional medical networks. The professional networks were also provided the option of sharing the study invitation and registration link via newsletter and/or social media platforms; (ii) snowball sampling, whereby recipients of email invitations were encouraged to share with other eligible HCPs, outside of the member networks; and (iii) promotion via social media platforms X (formally known as Twitter), LinkedIn and Facebook, including paid advertisements on the latter in Arabic and English. Advertisements in Arabic via Facebook targeted HCPs within the Arabic-speaking community in Australia, who may have a deeper understanding of community perspectives. A reminder invitation was sent through all professional network mailing lists and posted onto social media platforms one week before registrations closed.

### Data collection

Focus groups and interviews were conducted online between April and November 2023. The online discussion was audio recorded and went no longer than 60 min. A total of five focus groups were planned, each comprising three to eight participants to ensure diversity of perspectives while allowing for in-depth discussion within groups. A fixed sample size was not determined a priori; instead, recruitment continued until thematic saturation was reached, defined as the point at which no new codes or concepts emerged from successive focus groups [33].

Each focus group/interview was facilitated by a minimum of two investigators, with one leading the discussion (TL, AP) and a second who took notes (RSM, MG). RSM, an Arabic-speaking investigator, was present at each focus group/interview, who took notes and provided cultural insights where necessary. The facilitators were oncologists (AP) or researchers (TL, RSM) with expertise in cancer clinical trials (AP, TL, RSM) and/or qualitative research (TL, AP, RSM, MG). Participants were informed that the facilitators were independent researchers from the University of Technology Sydney (UTS) or South Western Sydney Local Health District (SWSLHD).

Due to the recruitment strategy, one facilitator (AP) had a prior professional relationship with some participants. This relationship is acknowledged and may have influenced the focus group dynamics. Efforts were made to ensure all participants felt comfortable sharing their perspectives freely. This relationship was acknowledged and managed through reflexivity and transparency in the research process. Their participation was voluntary and based on their professional experience relevant to the study topic. These participants later contributed to manuscript preparation by reviewing the interpretation of the findings and providing feedback. Their dual roles as both participants-come-reviewers of the analysis are acknowledged and considered in the interpretation of results.

### Analysis

**Fig. 1:**
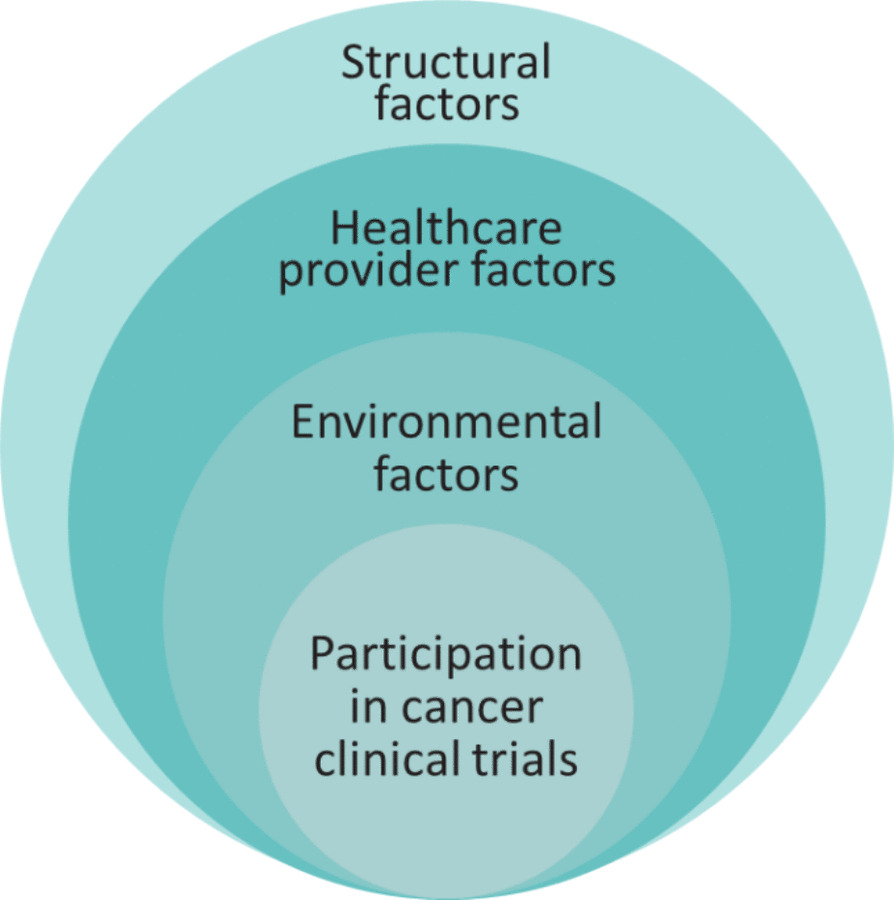
Coding framework—categorising upstream SDOH impact on participation in cancer clinical trials

The audio-recordings were professionally transcribed and imported into NVivo 14 Pro [34] and analysed using the “Best Fit” method. The Best Fit method was chosen as it combines both deductive and inductive approaches to analysis and is well suited to applied qualitative research. While themes were initially developed inductively through close engagement with the data, a pragmatic decision was made to use an a priori sociocultural framework to organise and interpret findings in a way that supported translation to equity‑focused clinical trial practice [35]. A coding team of two investigators (LE and RSM) independently familiarised themselves with the data and coded an initial subset of transcripts, then met to discuss. LE has expertise in population health with a focus on structural influences and RSM co-facilitated all focus groups/interviews. A coding framework was developed collaboratively and refined as analysis progressed before alignment with the predefined categories of the Sociocultural Framework for Health Service Disparities (see Fig. 1):

*Structural barriers/enablers*: refer to factors embedded within the study site (i.e., policies or practices) or trial sponsor that either obstructed or facilitated the participation of ASCPs in cancer clinical trials.

*Healthcare provider barriers/enablers*: refer to roles that persons providing healthcare services, especially those related to the role of recruiting clinicians, play in shaping clinical trial participation.

*Environmental barriers/enablers*: refer to social factors or personal experiences (throughout the life course) perceived to influence choice and behaviour via either encouraging or preventing participation in cancer clinical trials. HCPs based these perceptions on their experience recruiting ASCPs into cancer clinical trials.

Reflexivity was maintained through memo-writing and ongoing team discussions. Regular meetings were held to reflect on how the coders’ background and expertise influenced interpretation of the data. Once all transcripts were analysed, coders met to review and refine the categories to enhance consistency. Categories and emergent subthemes, supported by exemplar quotes, were presented to the full investigative team, who provided additional insights and nuance that informed interpretation. This analytic approach enabled in-depth interpretation of participants’ perspectives while also supporting the generation of findings relevant to policy and practice.

### Ethics

Ethical approval for this study was granted by the UTS Human Research Ethics Committee (HREC) (ETH21-6506, 14/01/2022). All participants gave informed consent to participate. Transcribed data were de-identified and securely stored in a restricted access folder on a UTS server. Only one investigator (RSM) had access to the recorded focus groups and interviews to protect participants’ confidentiality and privacy. All identifying data (e.g. names or workplaces) were removed from the transcripts and not available to the wider research team.

## Results

Fourteen HCPs (eight female and six male) participated in the study including focus groups (*n* = 13) and semi-structured interviews (*n* = 1). Ethnic background included: North African/Middle Eastern (*n* = 4), South-East Asian (*n* = 3), Northwest European (*n* = 3), Southern/Central Asian (*n* = 3), and one from an Oceanian background. English was spoken by all participants; three also spoke Arabic while four spoke a language other than English and Arabic. Participants were from New South Wales (*n* = 11) and Victoria (*n* = 3), predominantly working in urban healthcare settings (*n* = 11), followed by regional (*n* = 2) and rural (*n* = 1).

Participants represented a range of professional backgrounds, including medical oncologists (*n* = 7), radiation oncologists (*n* = 1), palliative care physicians (*n* = 2), and one each Cancer Care Coordinator nurse, oncology nurse, clinical trial coordinator and researcher. Experience recruiting ASCPs into cancer clinical trials ranged from 3–10 years’ (*n* = 6) to 11–20 years’ (*n* = 3), with two HCPs reporting greater than 20 years’ experience; three HCPs did not provide such details.

The perceived barriers and enablers reported by HCPs were thematically organised into structural, healthcare provider and environmental categories and subthemes. Table 1 summarises the findings and provides exemplar quotes.

**Table 1:** Barriers and enablers to cancer clinical trial participation among Arabic-speaking cancer patients

	**Barriers**	**Enablers**
**Structural factors**		
*Clinician consultation time*	Standard consultation times are insufficient to facilitate trial discussions with ASCPs *Just to [discuss] standard care treatments* [with ASCPs] *and so doing a clinical trial on top of that is a step too far when you’ve already spent 35 min in a 15-min consult.* (PID4.5_Specialist clinician) This was seen to influence whether clinicians would have trial discussions during consultations with ASCPs *The problem is the clinician will not say this out loud, we will just bypass* [an ASCP] *because* [the clinician] *knows… it’s a 15-min appointment* [and think] *Oh no. Yeah, no way. I’m going to recruit the* [Arabic-speaking] *patient here* (PID1.2 Specialist clinician)	HCPs cited booking longer or follow-up consultations with ASCPs and family members to ensure adequate understanding *In my experience with Arabic-speaking populations getting rapport, understanding the initial hesitations about treatment, about the diagnosis. Going through the diagnosis, going through what the stage is, what the diagnosis is, and you know having that conversation, it can take, you know, a couple of sessions in itself with family members… Then clinical trials tends to come up at that end of things,* [if] *they’re still well.* (PID2.1_Specialist clinician)
*Resource availability*	Lack of translated materials *I think we have two big barriers that we can see. One of them, mainly the language barrier and actually having information that’s available in Arabic, written in Arabic. And that goes even beyond research. Even part of the standard of care. So, providing written information for consent for treatments*. (PID2.3_Specialist clinician) Poor access to interpreters *We also have a very good trials team that are happy to see the patient after we’ve seen the* [Arabic-speaking cancer] *patient, but we’ve just had so much difficulty booking an* [Arabic-speaking] *interpreter for the discussion.* (PID4.3_Specialist clinician) Online interpreters were less preferred [Having an interpreter] *face-to-face is ideal but I’m pretty happy with the way that most telephone conversations go… I don’t find that dynamic with my virtual care quite as good cause… you’re in a triangle, you know, with the patient. You’re looking at the screen. The patient’s looking at the screen, and you’re not actually talking to the patient. You’re talking to the… camera, you know, so it’s very hard in that context to be, you know, keeping an eye on the camera, keeping an eye on the* [Arabic-speaking] *patient in the room. But that dynamic there is certainly different.* (PID5.6_Specialist clinician) Reliance on suboptimal interpretation *I think I’ve had… challenges where an interpreter, no matter how nuanced the conversation I’ve held or how information dense it’s been that’s been filtered and you get a one word or incorrect organ, and you only learn when you’ve got an English-speaking son or daughter present and they’re shaking their head because that hasn’t been conveyed appropriately.* (PID4.7_Specialist clinician)	Resources addressing general research concepts (e.g. randomisation), beyond information specific to a single trial, were considered useful in supporting recruitment *So, if we think about resources that might support that* [recruitment] *in the context of trials, it could be that there’s… a list of priority aspects that absolutely must be communicated. You know, the voluntary nature of it, the, you know, the randomisation, as we’ve discussed and things like that*. (PID2.3_Specialist clinician) HCPs advocated for translated resources *… my consistent message for quite some time now has been we need materials in other languages. It’s just not, you know, it’s not OK to have a conversation about a treatment. And all I can provide them* [ASCPs] *with is an English written piece of information. It’s not that hard for them* [i.e., study sponsor/drug companies] *to get a translated version and so we’ve engaged. I’ve engaged personally with about three companies now and they’re actually coming on board to provide us with* [translated] *material.* (PID2.3_Specialist clinician) HCPs booked longer consults and engaged interpreters in lieu of translated materials to enable trial discussions *You know, it was not going to happen to get a PICF translated into Arabic. So, it was more a case of getting help and interpreter to sit down and go through the PICF and book a one-hour consultation with a face to face* [interpreter] *but you know what, eventually that patient didn’t consent to proceed. And I suppose in the best way it was good because I wouldn’t have been able to deliver the same level of care and get informed consent to all the procedures that came with that trial… I wasn’t going to get any written information translated.* (PID2.1_Specialist clinician) Good access to interpreters *I think* [we have] *about 5 in-house Arabic Interpreters. So, accessing interpreters certainly is not as difficult for Arabic* [speaking cancer patients] *as compared to some of the languages within our area, and if that fails, we’ll always have the option of phone interpreter, although that’s not as good, but for trial purposes we would always have a qualified interpreter.* (PID2.3_Specialist clinician)
*Current research process*	Current trials exclude potential participants who have low-to-no English-language proficiency *Many of the clinical trials, they will only recruit few people who speak English anyway, and exclusion criteria will be always not native speakers. So… that will make it another barrier from the other end for* [Arabic-speaking] *people to look to be recruited in trials.* (PID1.2_Specialist clinician) HCPs highlighted that study sponsors would only translate study materials for language groups with a larger representation *I’ve come across one patient at* [study site] *where I requested a PICF* [participant informant and consent form] *in Arabic and that was like requesting a Unicorn. You know, it was not going to happen to get a PICF translated into Arabic. I put it back to the sponsor of the trial. Why can’t we get translations? It’s because of the numbers they said. If you had lots of patients who spoke that language that could justify us paying for translations… You’re almost set up for failure.* [ASCPs] *are set up to never to even consider a trial or have the resources or time to consider the trial.* (PID2.1_Specialist clinician) Challenges in translating scientific terminology *So, the word “trial” sometimes sounds like experimentation. We’ve had prisoners of war that have resettled in Australia, and so they see that as experimentation and therefore will decline to participate in everything that’s got a “trial” on it. And that also extends to things like drug access programmes. So sometimes you get a medication that’s not PBS listed but provided for free by the company until the PBS listing kicks in. I’ve had* [Arabic-speaking] *patients decline that as well because they feel this* [is an] *experiment.* (PID2.3_Specialist clinician) Lack of culturally competent translated materials and use of medical jargon adds burden on family supports *I feel like the family* [is] *also overwhelmed… there’s a lot of things that is scientifically hard to translate. Even though family can support… I could see the daughter was very anxious and stressed. I think it is common for patients…* [to be] *under a lot of already stress of not knowing what’s going to happen and plus the* [scientific] *language makes it more, I think, stressful.* (PID1.3_Research role)	Simple language for informed consent *The consent it is meant to be written in a very simple way. I know this because, you know, it’s really hard to understand the protocol. So you have to make the consent to be written in a very simple and easy way for patients to understand and family as well*. (PID1.2_Specialist clinician)
*Workforce diversity*	Lack of workforce diversity impedes research participation *I did have someone that… could speak Arabic. That’s certainly not a certified translator, but I don’t have that person on my team anymore. And so it makes it hard for us to then try and include that in the budget* (PID3.2_Research role)	Workforce diversity enables trust at individual/family-level *I think I know also it’s easier for* [Arabic-speaking] *patient to trust you, especially when you come from similar backgrounds… [they] trust you.* (PID1.2_Specialist clinician) *I find it like,* [Arabic-speaking] *people get more comfortable when they can hear someone speak their language 100%.* (PID1.3_Research role)
**Healthcare provider factors**		
*Principles of equitable access*		HCPs understood the importance of equitable access to trials *We just have a responsibility to ensure that people are given equal opportunity to take part in research. Yeah, that there’s equity and access to medical care and you know… access to clinical trials provides, I guess you could say, more oversight [into] your medical care from your health practitioner, but it also gives you access to potentially medications that are not approved for use in that indication. So, you know, you have the opportunity to have access to medications that you may not through, you know, your general sort of healthcare, but also access to combinations of medications* (PID3.2_Research role) Cultural competence *So, from my perspective, language is not a contraindication. It doesn’t rule you out. It just means that we need to ensure there is an interpreter available to have the discussion for signing of consent and for trial related procedures*. (PID2.3_Specialist clinician)
*Language discordance*	Language barriers *What I think is really useful when you’re speaking to patients who can speak your own language, you get immediate feedback of how your communication style is working. So you just fine tune that communication to the individual patient, which is so much harder when you’re dealing with someone* [who is Arabic] *speaking or* [non-English-speaking]… *You know, so you’re not getting that that immediate feedback*. (PID4.5_Specialist clinician) *Look for me because as I said, I’m able to speak the same language, I’m able to establish that rapport, I’m able to explain* [the trial] *to* [ASCPs]*. I don’t need someone in the middle. Then I can say I’m 100% confident when* [the ASCP] *agrees* [to participate]*. They agree. Yeah, but will it be the same if I have to translate to someone who speaks Chinese? Definitely not.* (PID1.2_Specialist clinician) Language barriers reduced HCPs confidence in patients’ understanding *Then there’s the question of do they* [ASCPs] *understand the voluntary aspect of it* [the trial]*… Are they really consenting to something that they fully appreciate? Do they see benefit?* (PID3.2_Research role)	Interpreter engagement and family involvement in discussions *I insist on* [an interpreter]*. I think it should be standard of care regardless. So I insist on face-to-face as well and will insist the family members* [are present] *as well, to say this is this is a requirement. This is our standard unless they say we absolutely don’t want one… and they actively refuse. But I do insist on face-to-face that’s ideal. And it often happens, if not telephone interpreter. If not, a family member will translate.* (PID2.1_Specialist clinician)
**Environmental factors**		
*Family support networks*	Role confusion reducing patient autonomy *I think doctors always remind family that that’s a decision that a patient need to take. But you know when you don’t speak the language yourself as a doctor, you will not understand what’s happening over there. I saw it like myself, where like even a daughter wanted to sign the consent on the behalf* [of her mother]*.* (PID2.3_Specialist clinician) HCPs noted this was especially the case for older ASCP who did not speak English and had younger family supports. HCPs noted that decision making could be influenced by their beliefs *Every time you discuss a trial with someone who speaks only Arabic, most of the time there would be a family member, or more than one family member, and those are the ones who really are making the decision on their behalf. So the daughter and the son will be making the decision on the behalf of the father or the mom, because usually if they only speak Arabic, they’re gonna be the elderly people in the family… and then it becomes difficult because now we are negotiating something or telling them something. It doesn’t matter, like if they have the paperwork, whatever… the ones who are making the decisions are really the family members and it depends on their beliefs as well. So it’s again the autonomy, the patient here is kind of is a bit different. In that regard, and that again cannot fix the trust in a way.* (PID1.2_Specialist clinician) Reliance on family support system for transportation *A lot of the Arabic patients rely on family members to get to appointments, to get to the hospital, and they struggle to even sometimes show up for appointments when they’re relying on family members who are also working, got young families. So that’s another barrier, I guess.* (PID4.6_Nurse)	Strong family support systems *I’ve found that when they* [ASCPs] *present with family members who are also motivated about and invested in the patients care, I think that makes a little bit of a difference because they kind of take on that information and help interpret it and also kind of carry on those conversations after the consult has ended.* (PID4.7_Specialist) HCP understood the importance of the family support system thereby showing cultural competence *I think having a specifying openness to questions from family members. So just building that into acknowledge that family or next of kin plays a role in the decision making* (PID4.4_Specialist clinician)
*Scepticism*	HCPs perceived that the ASCPs and their family supports were sceptic of clinical care and trials *… Getting ASCPs to actually trust that we are sort of recommending therapies, treatments, trials you know for their benefit and not so much to benefit the hospital, the drug companies… those themes sort of keep coming back.* (PID2.3_Specialist clinician) Some HCPs noted that this scepticism may be the result of a history of disjointed care and distrust in medical services *We have a lot of, you know, humanitarian visa migrants that are coming over with very poor health literacy, very sort of fractured care across different countries having gotten through three different countries to get to Australia*. (PID3.2_Research role) *… I think it’s kind of a distrust of medical service that seems to be underlying* (PID4.5_Specialist clinician) In other cases, HCPs identified this scepticism may be the result of younger family supports looking to protect their elder family members *Getting the family on side is a really important part of the process… especially for older people. And that’s because of the sort of protective, the family becomes a sort of protecter, you know, protective role of the older person. And they want the best for the family. I understand that kind of things*. (PID1.3_Research role)	HCPs attempted to understand and mitigate the perceived scepticism by allowing more time for conversations about their decision *As I said, I try to reflect on…what could be my barriers* [to participation]*, what* [might] *stop me from explaining this* [the trial] *in the right way? What stopped them from joining the trial or accepting that treatment or whatever? And I only check for the understanding before I go ahead. And again, allow more time for* [ASCP] *to talk and for me to listen, so I can understand better and all their questions like… how it works in Lebanon, for instance… Can you tell me [it works] there? How clinical* [trials] *work in your country or how they… work in your family? And then they start to talk, and, for me, I start to understand more and through this we kind of develop similar background that I can build upon. And we start even advancing the* [clinical trial] *conversations*. (PID2.1_Specialist clinician) HCPs noted that when a ASCP and family support shared a previous negative experience they would listen in order to improve future care *They had their own complaints about the health system, about… some experience in the hospital at some stage and really like it was… they were very vocal about their experience in the hospital… So I felt like this another opportunity for me to engage and hopefully help to improve.* (PID1.2_Specialist clinician)
*Additional stress and worry*	Perceived additional distress among ASCPs *I think the main barrier* [for ASCPs] *is the anxiety about the diagnosis, anxiety about the news of progression, that anxiety that the treatment has stopped working. That unaddressed anxiety about this, you know, horrendous disease, even though sometimes it’s a chronic disease and can be controlled, you know with oncology head on, it’s still viewed as still such a disastrous thing that unless you address that anxiety…Then you can’t move on with everything else you know and get informed, enthusiastic, or calm consent to carry on.* (PID2.1_Specialist clinician) Distress extended beyond patients, with HCPs noting similar emotional strain among family supports *I think it’s more the anxiety of the family more than the patient, you know, sometimes I feel because he’s* [the son] *attending on behalf of his mom and like the mom is not there. So I think it’s too much sometimes*. (PID1.3_Research role) *I find the anxiety a lot of it focuses on prognosis in* [ASCPs] *which is to not tell the patient the prognosis when it’s an elderly relative. That’s very much a recurring theme… if we tell them how bad it is, they will just give up and not accept treatment. That can be quite challenging.* (PID2.3_Specialist clinician)	

*ASCP *Arabic-speaking cancer patient, *HCP *healthcare professional, *PICF *participant information and consent form, *PID *participant identification number

### Structural barriers and enablers to cancer clinical trial participation

At the structural level, four subthemes emerged from discussions with HCPs: clinician consultation time, current research process, resource availability and workforce diversity (Table 1).

#### Clinician consultation time

Clinician consultation time was a major and ongoing structural-level factor that could both prevent and enable trial participation. Many HCPs cited that standard clinician consultation times were “15-min” in duration which were not long enough to discuss and recruit ASCPs into clinical trials. Many HCPs cited that recruiting clinicians needed “double the amount of time” or multiple consultations to recruit ASCPs into clinical trials compared to other patient groups. HCPs explained this was necessary to ensure that ASCPs, and their family supports, were given enough time to understand the context of the trial, ask questions, and the need for an interpreter (and to account for stop-start conversations).

Standard consultations were also observed to influence recruiting clinician practices. In some cases, HCPs noted they booked longer, or follow-up consults to mitigate the issue of insufficient time. However, one HCP cited that clinicians might overlook recruiting an eligible ASCP due to this time limitation.

#### Resource availability

Most HCPs noted that the lack of translated trial materials impeded research participation. HCPs cited that study sponsors had not previously provided translated materials due to ASCPs being a small representative group and therefore did not allocate, or could not justify, a budget to that identified need. This was also observed to extend beyond clinical trials and was an issue in providing standard cancer care. In lieu of translated materials, many HCPs became enablers to research participation by advocating on behalf of ASCPs for these materials, they booked longer consultations to go over trial information and ensured interpreters were available to assist ASCPs read through English-printed trial information.

Whilst some HCPs reported good access to Arabic-speaking interpreters, others experienced difficulties accessing an interpreter which limited trial and consent discussions. There was also an observed preference for using face-to-face interpreters. Some HCPs cited face-to-face enabled more effective communication compared to online or phone interpreter services. One HCP noted that whilst they might not understand the conversations between interpreters and ASCPs, they were able to gauge patient understanding via body language, which assisted their confidence in the ASCPs’ understanding of the trial and consent process. However, some HCPs indicated that poor interpretation of information could create barriers to trial participation. For example, one HCP described how, in their experience, family members in the room who spoke both Arabic and English disagreed with the interpreter’s interpretation of the trial information. Moreover, another HCP also noted that engaging interpreters during recruitment alone was insufficient to retain ASCPs in a trial, as ongoing support was needed to explain procedures throughout, posing an additional barrier in the research process and retention of ASCPs beyond recruitment.

#### Current research process

The current research process at a sponsor level (i.e. trial design) revealed several immediate barriers to clinical trial participation among ASCPs. HCPs noted that many clinical trials restrict recruitment to English-speaking/proficient cancer patients, thereby automatically excluding ASCPs with limited to no English proficiency. Whilst poor access to translated materials has been previously described, an additional “hurdle” to obtaining translated materials was the process of getting translated documents approved for use by the relevant ethics committee.

HCPs noted that ASCPs face similar challenges to the general population in understanding complex trial concepts, such as randomisation and placebo, with added difficulty due to the lack of translated trial materials. Many HCPs also noted that certain scientific terminology and concepts did not translate appropriately into Arabic. For example, “the word trial sometimes sounds like experimentation”, leading to misunderstandings and/or misconceptions about the purpose or integrity of the trial. This contributed to a lack of trust and scepticism, in turn discouraging ASCPs from participating in cancer clinical trials. Moreover, some HCPs noted that, in some cases, this mistrust extended beyond research to impact other aspects of routine medical care.

Moreover, challenges in translating scientific terminology were observed to have downstream effects on both ASCPs and their family caregivers. One HCP noted that the use of scientific language, coupled with the reliance on family caregivers to interpret and translate this information “overwhelmed” both ASCPs and their family support network. This added burden could act as a barrier to clinical trial participation.

#### Diversity in the workforce

Diversity in the workforce was identified as an enabler of trial participation. Arabic-speaking and other ethnically diverse HCPs noted that when healthcare teams, particularly recruiting clinicians, spoke/understood Arabic, it promoted trust and could enable the HCPs to effectively recruit ASCPs onto clinical trials.

### Healthcare provider barriers and enablers to cancer clinical trial participation

At the healthcare provider level, two subthemes emerged: equitable treatment and confidence in patients’ understanding.

#### Principles of equitable access

All participating HCPs acknowledged that all patients should be considered and offered a clinical trial regardless of their language proficiency, ethnic or cultural background, demonstrating an understanding of equitable access to cancer clinical trials. By adopting this mindset, HCPs themselves became enablers of research participation. Many HCPs noted strategies that mitigated structural barriers, such as allowing more time (or booking multiple consultations) and having an interpreter organised for face-to-face consultations, as previously described. Other strategies cited include rapport building and advocating for, and actively requesting, translated materials from the appropriate authorities. Moreover, HCPs demonstrated cultural awareness by understanding the collective approach to decision-making among ASCPs, whereby an individual may want to consult with their family support network and accounted for this in their consultations by including family in the discussion.

#### Confidence in patients’ understanding

Non-Arabic-speaking HCPs reported that language discordance with their ASCPs made it difficult to communicate trial information effectively, leaving them uncertain whether their ASCPs fully understood the scope of the clinical trial and/or the consent process. Whilst HCPs had previously described engaging interpreters to help overcome this barrier, many also cited challenges with interpreter access and/or quality of service. In some cases, engaging an interpreter service did not improve the HCP’s lack of confidence in patient’s understanding. Arabic-speaking HCPs indicated that while they were confident in their ASCPs’ understanding, they acknowledged that if they were recruiting patients from other non-English-speaking or non-Arabic-speaking communities, they would likely experience similar concerns.

### Environmental enablers and barriers to cancer clinical trial participation

Three overarching environmental subthemes emerged: family support network, scepticism, and additional stress and worry.

#### Family support network

HCPs observed that ASCPs had a strong sense of family and social support network. Some HCPs perceived family support as an enabler to trial participation by facilitating the interpretation of trial information and carrying on those conversations beyond the consultation. However, in other cases, some HCPs perceived family involvement as a barrier, whereby family members sometimes overstepped their role and became decision-makers, hindering patient autonomy. One Arabic-speaking HCP stated, “We don’t have that patient autonomy, it’s a family decision”*.* This was observed to be an issue particularly among elderly ASCPs, whereby their younger supports would make decisions on their behalf. Most HCPs described how decision making was a group decision among this patient group and would facilitate this by including all people in the room to ask questions. However, in some cases, HCPs noted that ASCPs relied on their younger family members not only for emotional and decision-making support but also for practical assistance, such as transportation. This reliance could impede not only clinical trial recruitment and retention but also continuity of routine medical care.

#### Scepticism among ASCPs and their family supports

Some HCPs described scepticism among ASCPs and their family supports as though it was a culturally normative response. The perceived cultural norm of scepticism and resulting mistrust extended beyond clinical trials to encompass attitudes towards cancer diagnosis and the Australian healthcare system more broadly. In the context of cancer clinical trials, some HCPs perceived that ASCPs were sceptical of the potential benefits of participating, leading to mistrust about the purpose of the trial. In other cases, they found that this mistrust may have stemmed from disjointed medical care originating outside of Australia. For older ASCPs, HCPs noted that younger family supports may be sceptical of trial discussions for the same reasons, or they wanted to protect their elder ASCP. To mitigate this barrier, HCPs, including Arabic-speaking HCPs, would try to build rapport; to understand ASCPs’ reservations towards trial participation by allowing more time to understand the cancer care journey and for questions. This highlights how HCPs themselves can be enablers of cancer clinical trial participation.

#### Additional stress and worry

Similar to above, some HCPs implied that ASCPs and their family supports commonly experienced higher levels of “*stress*” and “*anxiety*”, which were framed as culturally embedded responses, compared to other patient populations. This heightened sense of worry, coupled with the challenges of understanding, scepticism, and coming to terms with their cancer diagnosis, was cited as an additional barrier to trial participation and as a factor influencing HCPs’ readiness to initiate discussions about clinical trials.

## Discussion

This study explored upstream barriers and enablers of cancer clinical trial participation among ASCPs from the perspective of HCPs. To our knowledge, this is the first study that has looked beyond individual-level factors (such as age, sex, education etc.) to examine how upstream SDOH can influence ASCPs’ participation in cancer clinical trials.

All HCPs agreed that spoken language should not determine access to clinical trials. However, nearly all identified barriers (structural, healthcare provider, environmental) were linked to English-language proficiency. Language eligibility requirements were explicitly described by HCPs as exclusion criteria for some clinical trials; however, participants also emphasised that the underlying barrier was not limited English proficiency itself, but insufficient time, resources, and workforce diversity to address language needs, reflecting broader systemic gaps in cultural competency. Equitable access to healthcare means that everyone, irrespective of their background, age, English-language proficiency, socioeconomic status, geographic location, and other factors, has the capacity to access essential health services when and where they need them [36, 37].

In this context, ASCPs were often not given the opportunity to consider trial participation, undermining autonomy of choice. These structural constraints also shaped clinician behaviour, with recruiting clinicians less likely to approach ASCPs due to limited system-level support for inclusive recruitment. Such gatekeeping highlights how system- and healthcare provider-level factors influence which patients are informed about or invited to participate in clinical trials. A lack of opportunity, driven by limited access to and/or awareness of trials, is consistently identified as a key barrier to clinical trial participation among ethnically and culturally diverse communities [38]. Consistent with these findings, existing literature shows that English-language eligibility criteria are frequently embedded in clinical trials without justification, raising ethical concerns related to justice and contributing to disparities in access to trials and evidence-based care [39].

These structural barriers may have implications beyond cancer clinical trial participation, as HCPs described ASCPs might face broader systemic obstacles to receiving standard cancer care, accessing cancer-related services and resources, and building trust with healthcare providers, indicating systemic cultural incompetence. The literature highlights that a lack of culturally competent care can lead to poor health management such as late or misdiagnoses, treatment errors, and poorer health outcomes (such as pain palliation) among ethnically and culturally diverse communities [40–42]. Whilst these outcomes were not discussed by HCPs in this study, they might provide insight into the observed stress and worry, and/or scepticism ASCPs and their family supports were perceived to have towards cancer clinical trial participation more broadly. These findings are consistent with previous studies that demonstrated structural factors such as inequitable treatment [43] and language and interpretation issues [44, 45] can contribute to a sense of mistrust in the healthcare system. Moreover, unmet information needs (e.g. a lack of translated cancer-related materials) have been cited to exacerbate anxiety among Arabic-speaking communities [46]. At the healthcare provider-level, the proactive engagement of interpreters and advocacy for translated resources described by HCPs in this study, reflect a growing recognition of linguistic diversity as a core element of culturally competent care. Furthermore, HCPs demonstrated cultural competence extending beyond communication to encompass a nuanced understanding of family dynamics which were perceived as central to ASCPs’ engagement with healthcare and research. Together, these findings highlight the need for strategies addressing intersecting structural-, healthcare provider- and environmental-factors that influence ASCPs’ cancer clinical trial participation.

The current process in which informed consent is obtained was also highlighted within structural and environmental influences. Informed consent is a foundational ethical and legal process in healthcare and research, ensuring that individuals understand and voluntarily agree to medical treatments or participation in trials. It requires that the person giving consent is competent, receives adequate information about the treatment or trial, including its risks, benefits, and alternatives, and is free from coercion or undue influence [47, 48]. However, limited study resources (e.g. translated materials, interpreters) and role confusion, where family members influenced trial participation, undermined HCPs’ confidence in their ASCPs’ comprehension and ability to make an autonomous decision. This raises concerns over current informed consent procedures among ASCPs, with broader implications for other culturally and ethnically diverse populations with limited to no-English proficiency. Moreover, family members often act as informal interpreters, or as cited they might disagree with the formal interpretation. As a result, family members may feel responsible for bridging this language gap, which can either encourage or discourage the patient from engaging fully or consenting to trial participation [49, 50]. These factors might explain or add to the “stress” and “anxiety” both ASCPs and their families were perceived as having that prevented trial participation. Informed consent processes frequently rely on standardised written materials that may not align with local language, cultural context, or communication styles, with limited adaptation to participant needs [51, 52].

### Future implications

Adopting a rights-based approach to healthcare recognises access to clinical trials as a fundamental element of equitable healthcare [53]. This approach requires healthcare systems and study sponsors to take responsibility for addressing the inequities created by language barriers that influence both recruiting clinician practice and autonomy of choice to trial participation, ensuring all people have equal access to trials and their potential benefits. Addressing the structural barriers identified requires study sponsors to design trials with inclusivity in mind, including early consideration and appropriate resourcing of strategies such as translated study materials and access to interpreter services, guided by the demographic profile of the target population and study setting. Moreover, all people should have the right to make informed decisions about participating in clinical research [54]. As highlighted by this study, this requires the time and resources (access to translated, culturally appropriate trial information and interpreters) to enable communications about potential risks, benefits, and alternatives. A more equitable consent process might include study sponsors creating “short form consent” in the participant’s language, combined with an oral explanation via a qualified interpreter and/or ensuring that materials can be culturally adapted using simple, clear language [55, 56]. However, as a recent review notes, more research is needed on effective interventions to support research participation among diverse populations [57]. Artificial intelligence (AI) is a new tool that could also be utilised to enhance the informed consent process. Recent studies have found AI to improve the readability and accessibility of consent forms, generate patient-friendly summaries, streamline communication, and create interactive tools such as chatbots and multiple-choice questions to assess comprehension [58–60]. These approaches might assist ASCPs with their information needs, potentially mitigating HCPs’ lack of confidence in patient understanding. AI in this context requires caution due to risks related to accuracy and trust. Any future application would need strong safeguards, regulatory oversight, and a clear role as a support to, rather than a replacement for, human communication.

While the ideal is that study sites (i.e., healthcare systems) only host trials where sponsors have proactively considered the patient demographic during pre-planning and ensured sufficient resources to address identified cultural and language needs, this may not be immediately feasible in highly multicultural settings such as Australia, the United Kingdom and United States. As an interim step, efforts should prioritise the development of translated and culturally adapted materials for groups where there is evidence of poorer outcomes or lower research participation, thereby moving incrementally toward more equitable trial practices. Co-designing studies with clinicians can help overcome barriers by incorporating mitigation strategies in the study design, thereby promoting more open and inclusive recruitment practices [61]. Moreover, extending co-design to include community members and engaging trusted local leaders can further enhance awareness of and access to open trials [38, 62]. Study sites can also support HCPs to actively recruit potential non-English-speaking participants by reducing administrative burdens such as engaging interpreters for each patient and integrating research recruitment into routine care [63].

Benefits resulting from any scientific research and its applications should be shared with society, as agreed by HCPs in this study. Accordingly, healthcare policies and future clinical trials must support HCPs in recruiting ASCPs and other culturally and ethnically diverse communities to ensure that trial benefits and generalisability are not limited to only a subset of the population. If sponsors or study sites are unable to address the barriers reported in this study, the ethical implications must be carefully considered, including whether it is equitable or defensible to proceed with opening the trial [64]. Responsibility for overcoming these barriers should be shared across multiple levels: study sponsors and sites have a duty to consider participant demographics in trial design and implementation, while healthcare systems and policymakers play a role in creating enabling structures. For example, policies could support longer consultation times to facilitate trial discussions with ASCPs (and other identified patient populations) and their families. Innovative funding models could be implemented so that payments for recruiting English-speaking participants include provisions to offset the additional costs needed to recruit non-English-speaking participants (e.g. by funding translated study materials). This could help ensure trial participation is more equitable.

Workforce diversity was identified as a key enabler in establishing trust and rapport between ASCPs, their family support and clinical teams. A potential strategy to enhance this is to implement a bilingual patient navigation service [65]. This service has been shown to bridge language discordance, build trust, and improve recruitment and retention rates in clinical trials with other non-English-speaking patient populations [66–68]. Providing bilingual patient navigation services, which can include educational and psychosocial support, can help participants navigate the complexities of clinical trials easing the time of recruiting clinicians, assist HCPs’ confidence in patients’ understanding, and potentially address perceived scepticism and reduce patients’ and families’ stress [69, 70]. Other system-level strategies include staff investment and training, community outreach and recruitment strategies, and accountability measures to support diversity and inclusion sustainability [71–73]. Together, these efforts can help embed and sustain equity and inclusivity as standard practice across health systems and clinical research.

Achieving this requires systemic change, support from government, funding bodies and research institutions, community engagement, infrastructure development, and policy reform addressing barriers for ASCPs and other culturally and ethnically diverse communities. Ensuring equitable access to research is essential for advancing overall health equity, leading to improved health outcomes and a reduction in disparities.

### Strengths and limitations

Our study examined how upstream SDOH influence cancer clinical trial participation among ASCPs in Australia that go beyond individual-level barriers and challenge the assumption that reasons for non-participation in trials are primarily patient-related. While conducted in Australia, the findings are potentially transferable to other high-income healthcare settings with similar clinical trial structures. The cultural diversity of both the investigators and participants strengthens the interpretation, translation, and broader impact of the findings. This study uncovered how language is at the core of the inequity of access and awareness, a responsibility that study sites and sponsors must address to ensure equitable access. As noted, one facilitator had a prior professional relationship with some participants which may have changed focus group dynamics. This may have introduced bias in their responses to the topic guide questions and therefore the interpretation of findings. However, we sought to minimise this by ensuring all focus groups were facilitated by two people, that all data were coded independently by multiple researchers and results were discussed with these participants-come-investigators and the broader research team.

The findings presented here are based on HCPs’ perceptions only and do not include the lived experience of ASCPs. To gain a holistic understanding of this issue, it is essential to integrate the perspectives of patients and their family caregivers, whose lived experiences provide invaluable insights into addressing these inequities effectively. To address this gap, a follow-up study with adults from Arabic-speaking communities with an experience of cancer and/or research is being conducted to further explore the barriers and enablers to cancer clinical trial participation among Arabic-speaking communities in Australia.

## Conclusion

Findings highlight how structural, healthcare provider and environmental determinants can create barriers to cancer clinical trial access and participation among ASCPs in Australia. Although many barriers were described in terms of language, they likely reflect a broader lack of cultural competency within healthcare and research systems. Addressing these barriers is essential to ensure equitable access to the benefits of medical research, which is fundamental for understanding the safety and efficacy of new treatments across diverse populations. Further research into strategies that integrate key enablers, such as time, workforce diversity, interpreter engagement and translated resources, is warranted to inform more inclusive healthcare systems and research design, with implications extending beyond the Arabic-speaking community.

## Supplementary Information

Supplementary Material 1.

## Data Availability

Not applicable.
